# RNA-sequencing based gene variants observed in patients with hyperlipidemia and premature coronary heart disease: A preliminary study

**DOI:** 10.1016/j.bbrep.2026.102466

**Published:** 2026-01-24

**Authors:** Wilanee Dechkhajorn, Kriengchai Prasongsukarn, Surachet Benjathummarak, Supachai Topanurak, Yaowapa Maneerat

**Affiliations:** a Department of Tropical Pathology, Faculty of Tropical Medicine, Mahidol University, Bangkok, 10400, Thailand; b Phramongkutklao Hospital and College of Medicine, Bangkok, 10400, Thailand; c Center of Excellence for Antibody Research, Faculty of Tropical Medicine, Mahidol University, Bangkok, 10400, Thailand; d Department of Molecular Tropical Medicine and Genetics, Faculty of Tropical Medicine, Mahidol University, Bangkok, 10400, Thailand

**Keywords:** Variants, Hyperlipidemia, Premature coronary heart disease, RNA-seq, NGS

## Abstract

Familial hypercholesterolemia (FH) is a genetic disorder characterized by markedly elevated low-density lipoprotein (LDL) cholesterol levels, which primarily progresses to premature or familial coronary heart disease (FH-CHD).

This cross-sectional study included healthy controls (N) and patients with hyperlipidemia (H), FH, CHD, and FH-CHD. We attempted to explore gene variants shared in H, FH and FH-CHD using next-generation sequencing tool. The RNA-seq transcriptome profiling from the whole peripheral blood (n = 3/group) were analyzed. The results revealed 15 intersected gene variants between the H/FH and FH-CHD groups. Aligning and mapping on the coding regions showed significant high-impact variants in 6 of the 15 genes including *MAFG*, *AKAP1*, *TLR5*, *CHUK, EMC10,* and *PLRG1.* The significant high-impact variations included frameshift variants in *CHUK* and *PLRG,* stop-gain variation in *TLR5* at the last intron, stop-lost variation in *EMC10,* and splice-acceptor and donor variants in *MAFG* and *AKAP1*, respectively. Pathogenicity scoring (ACMG Criteria) interpreted that these variation effects are predicted to lose the gene functions. Based on reference databases without any validation, these gene variations are probably linked to atherogenesis and CHD development.

Conclusively, our exploratory observed that *MAFG*, *AKAP1*, *TLR5*, *CHUK, EMC10,* and *PLRG1* variants had higher impacts and might be related to premature CHD development. Further classification and functional validation of these genetic variations should be considered for the feasibility of using these gene variants as contributory predictors of the FH-CHD risk in hyperlipidemia patients.

## Highlight

• Familial hyperlipidemia (FH) contributes familial coronary heart disease (FH-CHD).
• RNA-seq analysis was used to explore variants in patients with H, FH, CHD, and FH-CHD.
• The intersected variants in *MAFG, AKAP1, TLR5, CHUK, EMC10,* and *PLRG1* were selected.
• These variants may be related to atherogenesis and premature CHD development.

## 1 Introductions

Coronary heart disease (CHD) is a complex manifestation of atherosclerosis. The pathogenesis of the disease, which involves a long preclinical course, remains unclear. Various risk factors for CHD include dietary patterns, behaviors and lifestyles, for example, stress, smoking, high-fat food intake, decreased physical activity, inflammation, infectious diseases, abnormal endogenous blood constituents (such as lipids, lipoproteins, and coagulation proteins), adiposity, hypertension, and diabetes mellitus [1,2]. Moreover, genetic alterations are significantly associated with premature CHD.

Hyperlipidemia is an abnormal condition with high blood levels of lipoproteins or lipids, such as fats, triglycerides, cholesterol, and phospholipids, and is subdivided into two categories: primary (familial) and secondary (acquired) hyperlipidemia. Acquired hyperlipidemia is caused by other underlying disorders and lifestyles that cause alterations in plasma lipid and lipoprotein metabolism. In particular, hypercholesterolemia, characterized by high level of low-density lipoprotein (LDL) cholesterol, is one of the most prevalent risk factors in the progression of atherosclerosis and vascular disease involvement (e.g., CHD). Familial hypercholesterolemia (FH), a genetic disorder, is characterized by heritable and severely elevated levels of LDL-cholesterol [3] and mainly contributes to the development of premature cardiovascular disease (CVD), particularly premature or familial CHD (FH-CHD) [1,2]. Studies have reported that at least four genes related to sterol and lipoprotein pathways, including LDL receptors, apolipoprotein (apo) B, proprotein convertase subtilisin/kexin type 9, and the autosomal recessive hypercholesterolemia adaptor protein, are associated with cholesterol metabolism disorders [4,5]. Moreover, other roles of these proteins and associated receptors, or other genes linked to the FH-CHD development, should be investigated to evaluate the risk of developing this disease.

RNA-sequencing (RNA-seq) has been widely applied to detect gene expression in various tissues [6,7]. This technique also effectively detects transcriptome variants, such as single-nucleotide polymorphisms (SNPs) and short indels, in divergent tissues and species [[8], [9], [10], [11], [12]]. The present study aimed to explore intersected gene variants shared only between groups of hyperlipidemia, FH, and FH-CHD patients for their potential as interdependent predictors of the possibility of FH-CHD progression in hyperlipidemia patients. Next-generation sequencing (NGS)-based findings were used to determine if the selected variants had potential as markers for FH-CHD risk in patients with hyperlipidemia.

## 2 Material and methods

The corresponding author can provide all study materials, data, and analysis methods in this study upon reasonable request to repeat the results or simulate the procedures. In this cross-sectional study, male volunteers from Thailand, including healthy controls (N) and patients with hyperlipidemia (H), FH, CHD, and FH-CHD were enrolled. The RNA-seq or transcriptome data obtained from whole blood samples from each group (n = 3/group) were analyzed by NGS. The variant sequences found specifically in the H, FH, and FH-CHD groups were filtered to generate an intersecting gene variant profile. Using transcriptomics, we explored the genetic variants within genes that influence the development of CVD, as described previously [10].

### 2.1 Ethics approval and consent to participate

The present study was conducted at the Faculty of Tropical Medicine, Mahidol University, and approved by the Ethics Committee of Mahidol University (MUTM2017-025-01-2) and The Institutional Review Board, Royal Thai Army Medical Department (Q031b/59). Before recruitment, and after being informed of the study objectives, the volunteers completed informed consent forms.

### 2.2 Participants

**Fig. 1:**
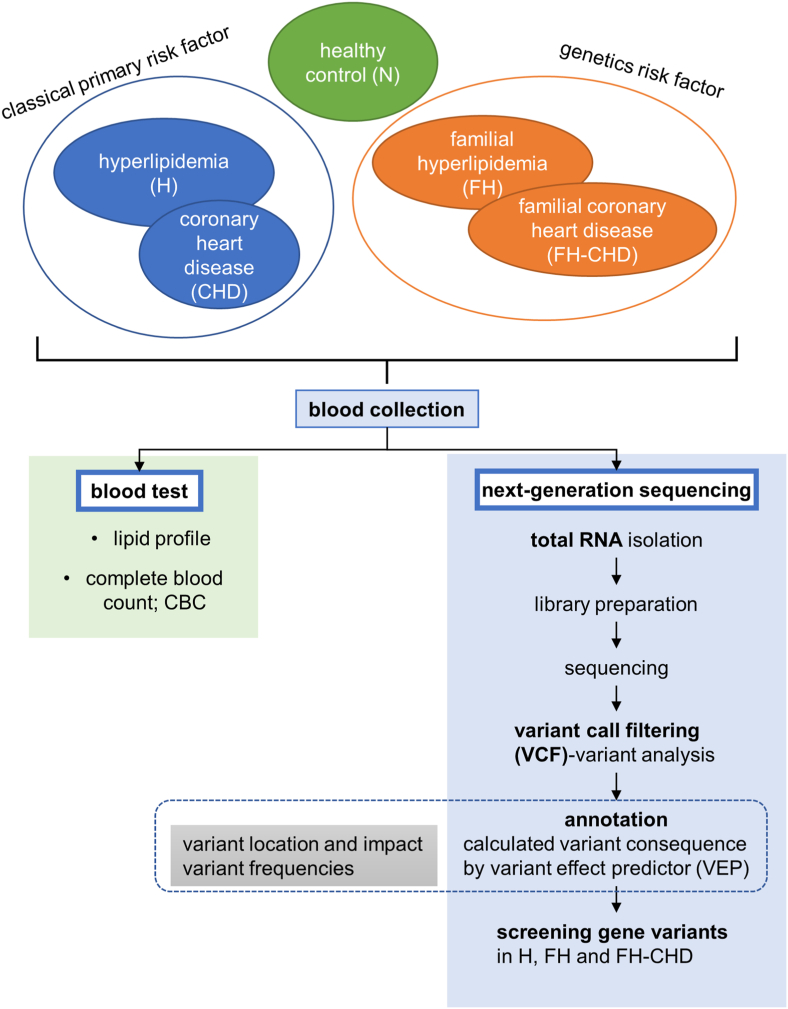
Experimental design and study population.

Fifteen male volunteers born to Thai parents were recruited in the present study and assigned to the five groups (n = 3/group). Healthy volunteers without any infectious and underlying diseases or CVD risk factors were enrolled as controls (the N group). The remaining patients (n = 12), who were diagnosed and treated by a specialist (KP) at Phramongkutklao Hospital, were classified into the following groups based on the American College of Cardiology/American Heart Association criteria (2013) [13] patients with high cholesterol levels [total cholesterol (TC) > 200 mg/dL, LDL >130 mg/dL, and high-density lipoprotein (HDL) < 40 mg/dL] but with no evidence of vital organ dysfunction (H group); CHD patients scheduled to undergo coronary bypass grafting (CHD group); and three pairs of family related patients with FH and FH-CHD. The FH patients were identified according to clinical criteria (including TC > 240, LDL >170 with or without tendon xanthomas and corneal arcus) and familial history. All H, FH, CHD and FH-CHD patients were treated with appropriate cholesterol-lowering drugs since they were diagnosed as prescribed by KP. The workflow of this study is shown in Fig. 1. The clinical manifestations of all volunteers are described in Table 1.

**Table 1:** Characteristics of the participants.

Variables	N *(n = 3)*	Hyperlipidemia *(n = 3)*	CHD *(n = 3)*	FH *(n = 3)*	FH−CHD *(n = 3)*
**Age** (years)	26 ± 5.1∗ (19−36)	56.7 ± 4.4 (50−65)	49.7 ± 4.5 (41−56)	38.3 ± 9.3 (20−50)	56.3 ± 11 (45−79)
**Lipid profile**					
Triglycerides (mg/dL)	108.3 ± 19.2 (83−146)	163.3 ± 28.3 (134−220)	245.7 ± 66.1 (141−368)	165.0 ± 45.5 (117−256)	19.0 ± 42.7 (125−271)
Total cholesterol (mg/dL)∗∗	174 ± 6.2 (165−186)	192.3 ± 24.6 (148−233)	160.3 ± 15.4 (131−183)	233.7 ± 32.0 (171−276)	193.0 ± 19.6 (171−232)
HDL-cholesterol (mg/dL)	48.3 ± 1.5 (46−51)	58.7 ± 9.9 (45−78)	44.7 ± 1.8 (42−48)	57.0 ± 12.4 (35−78)	45.3 ± 1.8 (42−48)
LDL-cholesterol (mg/dL)	104.0 ± 3.8 (98−111)	101.0 ± 31.9 (43−110)	66.3 ± 27.1 (15−107)	144.0 ± 27.6 (89−175)	109.7 ± 20.5 (80−149)
**Complete Blood Count (CBC)**					
WBC (10^3^/μL)	9.4 ± 2.1 (5.2−12.0)	5.9 ± 0.3 (5.6−6.5)	5.8 ± 0.2 (5.4−6.0)	6.8 ± 0.9 (5.4−8.5)	7.1 ± 0.9 (5.4−8.5)
RBC (10^3^/μL)	5.2 ± 0.4 (4.7−6.1)	4.3 ± 0.4 (3.6−4.9)	4.7 ± 0.1 (4.5−4.8)	5.6 ± 0.4 (5.1−6.3)	5.5 ± 0.7 (4.2−6.4)
Hb (g/dL)	14.8 ± 1.2 (13−17)	13.5 ± 1.1 (11.2−14.7)	13.7 ± 0.7 (12.5−15)	14.6 ± 0.2 (14.2−15)	14.3 ± 1.0 (13.3−16.2)
HCT (%)	43.2 ± 3.0 (38.6−48.8)	39.3 ± 3.2 (32.9−43.3)	40.1 ± 1.6 (38−43.1)	43.2 ± 0.3 (42.6−46.8)	43.3 ± 3.6 (38.3−49.7)
LYMPH (%)	37.1 ± 1.6 (35.4−40.2)	30.1 ± 5.1 (21.4−39.1)	32.8 ± 4.8 (23.3−38)	34.5 ± 6.5 (25−47)	30.1 ± 2.3 (27−34.5)
MONO (%)	4.9 ± 0.2 (4.6−5.2)	6.0 ± 0.3 (5.3−6.4)	6.1 ± 0.6 (5.3−7.3)	4.6 ± 0.3 (4−5)	6.6 ± 1.4 (4−8.7)

All patients and controls were male. Data are shown as the mean ± SEM (min-max).

N, normal controls; CHD, patients with coronary heart disease who were scheduled to undergo coronary bypass grafting; FH, patients with familial hypercholesterolemia; and FH-CHD, patients with familial coronary heart disease.

HDL, high-density lipoprotein; LDL, low-density lipoprotein; WBC, white blood cells; RBC, red blood cells; Hb, hemoglobin; HCT, hematocrit; LYMPH, lymphocyte; MONO, monocyte.

∗Age in N was significantly younger than H (*p* = 0.0106) and CHD groups (*p* = 0.0255),

while not significantly different from FH and FH-CHD groups (*p* > 0.05).

∗∗ LDL levels among all patient groups were not significantly different comparing to N group (all *p* > 0.05).

### 2.3 Blood sample collection and experimental design

Blood samples treated with heparin (5 mL) were collected from the all patients before hyperlipidemia treatment or coronary bypass grafting, as well as from healthy controls. Sera separated from 1 mL of clotted blood was used to detect lipid markers, including triglycerides (TG), TC, LDL, and HDL. Kits (Randox Laboratories Ltd., Crumlin, UK) and a biochemistry analyzer (Architect CI 16200, Abbott Laboratories, Abbott Park, IL, USA) were used to analyze the lipids enzymatically [14]. A QIAamp RNA Blood Mini kit (Qiagen Inc., Germantown, MD, USA) was used to immediately extract total RNA from approximately 2 mL of whole peripheral blood, which was then stored at −70 °C. High-throughput transcriptome sequencing was achieved by performing NGS, and the whole RNA-seq data were evaluated and mapped among the groups. Genetic variations found only within the coding regions of genes intersecting between the H/FH and FH-CHD groups were selected. The properties and functions of the selected variants were evaluated to find possible links with FH-CHD development.

### 2.4 cDNA library construction and NGS sequencing

Total RNA was collected from 15 samples (3 samples from each group). The amount and quality of all the total RNA samples were analyzed, and integrity was assessed using an Agilent 2100 Bioanalyzer. Only samples with a high RNA Integrity Number (RIN ≥8.0) were then subjected to cDNA library construction and NGS sequencing in duplicate, as described previously [14] at the Omics Sciences and Bioinformatics Center (Bangkok, Thailand). The statistical power of this experimental design, calculated in RNASegPower, was 0.53 [15].

### 2.5 Sequence alignment, variant identification, and annotation

Using bioinformatic analysis on whole RNA sequences, the variants were obtained, and their positions on genes were screened and filtered from all samples. Initial quality control of raw data files was performed using FASTQC software. Adapter sequence and low-quality reads were subsequently removed using Trimmomatic with the following parameters: ILLUMINACLIP: [AdapterFile]:2:30:10 LEADING:3 TRAILING:3 SLIDINGWINDOW:4:15 MINLEN:36 (specifying minimum base quality (Phred) of 3, minimum average quality of 15 over a 4-base window, and minimum read length of 36). The filtered reads were aligned to a human reference genome using the HISAT2 aligner. Variant calling was then conducted following the workflow outlined in Supplementary Method S1: Command Line Pipeline (Filtered_variants_called.vcf). Hard filtering criteria—such as “QD < 2.0” and “FS > 30.0”—were applied using GATK to obtain high-confidence variants [16]. The resulting variants were annotated with the Ensembl Variant Effect Predictor (VEP), which provided transcript-level consequences, affected genes, and functional classifications [17]. Additional biological annotation and relevant functional protein predictions were performed using NCBI (https://www.ncbi.nlm.nih.gov), GeneCards (https://www.genecards.org), and UniProt databases (https://www.uniprot.org).

### 2.6 Selection of gene variants focusing on coding regions share between H/FH and FH-CHD

The variant category counts and gene overlaps (intersected genes) were accessed from Venn diagram using InteractiVenn (web-based tool) [18]. Briefly, the VCF file (from 2.5) were annotated. Then only intersected gene variants sharing between H/FH, and FH-CHD groups focusing on high impact effect in coding region were selected. These variants were further determined properties, function and possibility associated with the progression of CHD based on reference databases.

## 3 Results

### 3.1 Characteristics of the patients and controls

The general descriptions and clinical manifestations of the H, FH, CHD and FH-CHD groups and the N group (n = 3/group) are shown as mean ± SEM (ranges, minimum-maximum values) in Table 1. The volunteers in the N group were significantly younger than those in the H (*p* = 0.0106) and CHD groups (*p* = 0.0255), while not significantly different from those in the FH and FH-CHD groups (*p* > 0.05). The age difference between the N group and the H and CHD groups is reasonable because the disease typically develops progressively with advancing age [1,2]. As all four patient groups received lipid-lowering medication, their LDL levels were not significantly different from those of the normal group (all *p*-values >0.05).

### 3.2 Preselection of the regulatory sequences

**Fig. 2:**
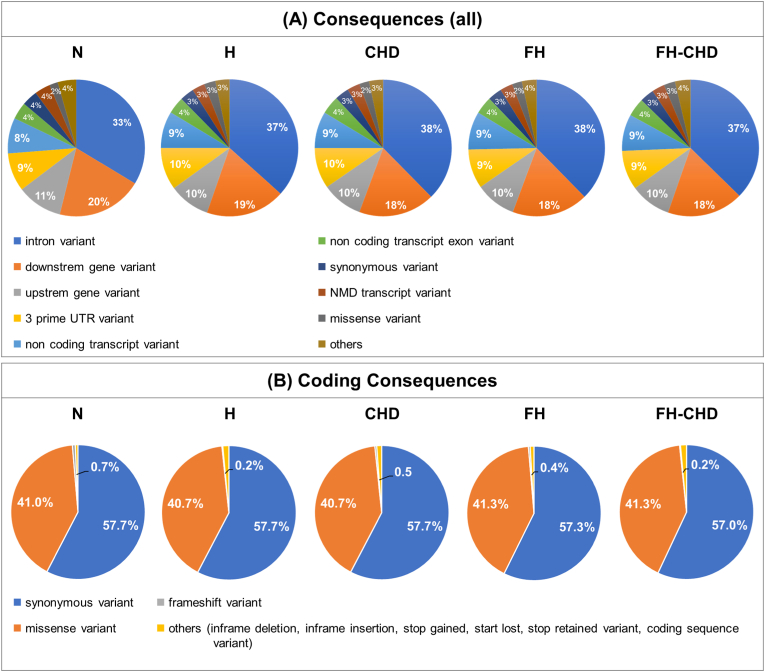
Gene variation annotation of the whole genome performed by transcriptome sequencing (RNA-seq**)** analysis. Four patient groups, hyperlipidemia (H), familial hyperlipidemia (FH), coronary heart disease (CHD), and familial CHD (FH-CHD) compared with healthy volunteers (N). (A) Panels show variations in the whole genome (all consequences); (B) panels show variants in the coding sequence (coding consequences).

**Fig. 3:**
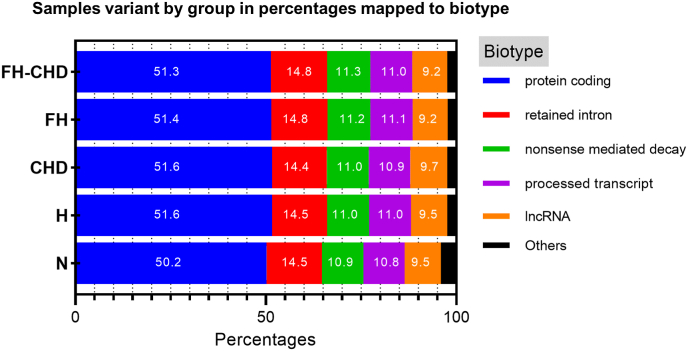
Sample variants by group in percentage mapped to biotype.

Several variants were screened from all samples in the N, H, FH, CHD, and FH-CHD groups by performing transcriptome sequencing (RNA-seq). The screening data revealed several variants in consequence (all) from all samples in each group. Focusing on the coding regions, the major variants included synonymous and missense variants. Minor variants included frameshift variants and in-frame deletions and insertions. The comparison between the number of significant gene variants in the four patient groups and the N group, the annotation analysis of all samples, and the severity of the impact are summarized in Fig. 2 and Table 2. Fig. 3 shows sample variants by groups in percentages mapped to biotypes, including protein coding, retained intron, non-sense-mediated decay, process transcript, IncRNA, and others.

**Table 2:** The counts of all variants of protein coding, and completely novel variants in each functional effect group separated by allele frequency category.

Consequence		N		H		CHD		FH		FH−CHD	
	Impact effect	variants	genes	variants	genes	variants	genes	variants	genes	variants	genes
3 prime UTR variant	MODIFIER	58211 (29725−111745)	5356 (4943−5905)	30230 (27682−34870)	5246 (4719−5999)	28762 (24822−34471)	5104 (4117−5976)	27451 (25775−28646)	4569 (4236−4864)	28234 (22061−32142)	4530 (3720−5305)
5 prime UTR variant	MODIFIER	8636 (5169−15269)	1657 (1585−1738)	4639 (3705−5318)	1478 (1236−1702)	4601 (3162−6103)	1464 (1074−1912)	4733 (4475−5040)	1500 (1448−1574)	4814 (3620−5711)	1526 (1163−1794)
coding sequence variant	MODIFIER	9 (5−15)	6 (5−8)	6 (4−7)	6 (4−7)	7 (3−−10)	7 (3−10)	9 (7−12)	9 (7−12)	9 (8−9)	8 (8−9)
downstream gene variant	MODIFIER	84069 (62783−126144)	6428 (6034−6929)	64652 (62059−69126)	6349 (5923−7077)	63203 (59837−67943)	6203 (5317−6912)	63411 (62725−63800)	5666 (5351−5929)	63468 (59047−66523)	5892 (4897−6467)
**frameshift variant^4^**	**HIGH**	**171 (98−276)**	**38 (33−44)**	**103 (77−123)**	**33 (27−40)**	**89 (71−112)**	**33 (31−36)**	**79 (58−109)**	**28 (22−37)**	**97 (86−116)**	**38 (30−46)**
In-frame deletion variant	MODERATE	207 (87−447)	28 (24−36)	95 (81−117)	24 (23−26)	85 (64−108)	23 (17−29)	87 (72−108)	24 (22−27)	79 (46−105)	24 (16−28)
In-frame insertion variant	MODERATE	151 (51−313)	18 (11−22)	61 (55−64)	15 (12−17)	63 (43−83)	14 (10−19)	68 (57−77)	16 (14−18)	63 (55−72)	15 (13−17)
intron variant	MODIFIER	164247 (88896−294635)	6041 (5853−6199)	109962 (96910−120324)	5959 (5741−6308)	113890 (95446−125819)	5984 (5270−6565)	114298 (111866−116473)	5511 (5273−5710)	112980 (100608−131380)	5752 (5101−6122)
missense variant	MODERATE	16736 (10013−29715)	2374 (2248−2501)	9161 (8238−10306)	2196 (1972−2481)	8782 (7008−11021)	2111 (1783−2612)	8990 (8369−9657)	13699 (1948−36950)	9133 (6827−10453)	2116 (1629−2482)
**splice-acceptor variant^1^**	**HIGH**	**37 (15−55)**	**12 (11−13)**	**28 (24−31)**	**15 (13−18)**	**32 (20−−43)**	**17 (12−19)**	**41 (23−58)**	**18 (14−21)**	**26 (25−27)**	**15 (14−15)**
**splice donor variant^2^**	**HIGH**	**53 (35−79)**	**14 (11−13)**	**22 (17−25)**	**12 (9−17)**	**24 (18−36)**	**13 (8−19)**	**20 (9−31)**	**11 (7−15)**	**25 (23−26)**	**12 (11−14)**
splice region variant	LOW	990 (618−1656)	225 (209−237)	628 (540−712)	214 (180−244)	622 (468−701)	206 (170−226)	628 (559−683)	218 (212−229)	2586 (659−6363)	232 (226−241)
**start-lost variant^6^**	**HIGH**	**31 (17−53)**	**11 (10−12)**	**22 (17−25)**	**13 (10−16)**	**20 (11−29)**	**13 (8−17)**	**17 (10−26)**	**9 (6−12)**	**34 (7−74)**	**10 (5−14)**
**stop-gained variant^3^**	**HIGH**	**44 (18−77)**	**14 (12−15)**	**17 (10−22)**	**11 (7−14)**	**23 (15−34)**	**12 (10−15)**	**19 (17−20)**	**12 (11−14)**	**20 (6−32)**	**10 (6−13)**
**stop-lost variant^5^**	**HIGH**	**14 (9−20)**	**8 (7−10)**	**11 (9−12)**	**9 (7−10)**	**11 (5−15)**	**9 (5−11)**	**8 (7−9)**	**7 (7−7)**	**9 (6−12)**	**9 (6−13)**
stop-retained variant	LOW	14 (7−20)	6 (5−8)	10 (5−16)	5 (4−7)	11 (9−−15)	6 (5−8)	9 (5−12)	6 (3−8)	13 (8−22)	7 (5−11)
synonymous variant	LOW	23129 (13736−40435)	2982 (2790−3257)	13116 (11697−14860)	2813 (2511−3210)	12370 (10054−15625)	2660 (2275−3317)	12717 (11811−13463)	2631 (2471−2724)	12671 (9998−14234)	2680 (2044−3063)
upstream gene variant	MODIFIER	37820 (25267−60500)	3730 (3654−3833)	27184 (26030−28293)	3603 (3374−3869)	27659 (25720−29509)	3618 (3354−3970)	28292 (27391−29657)	3502 (3449−3537)	29047 (28144−30060)	3561 (3128−3907

All data are shown as the mean (min-max).

∗1–6 are shown in order of severity (more to less severe) estimated by Ensembl as high impact.

### 3.3 Selection of intersecting target genes

**Fig. 4:**
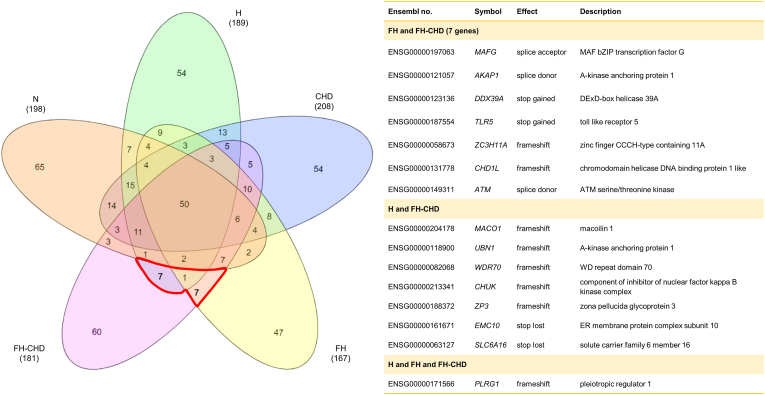
Venn diagram illustrates the variants focused on coding regions in the N, H, FH, CHD, and FH-CHD groups using transcriptome sequencing (RNA-Seq) analysis. The variant found in fifteen genes intersected between FH and FH-CHD (7 genes including *MAFG*, *AKAP1*, *DDX39A*, *TLR5, ZC3H11A*, *CHD1L*, and *ATM*), H and FH-CHD (7 genes including *MACO1*, *UBN1*, *WDR70*, *CHUK*, *ZP3*, *EMC10* and *SLC6A16*), H and FH and FH-CHD (1 gene is *PLRG1*) were selected for determining as feasible associate for FH-CHD risk.

The variants within the coding regions of the genes in all groups were assessed as illustrated in the Venn diagram (Fig. 4). We selected 15 intersecting variant genes with a high impact: *MAFG*, *AKAP1*, *DDX39A*, *TLR5*, *ZC3H11A*, *CHD1L*, *ATM, MACO1, UBN1, WDR70, CHUK, ZP3, EMC10, SLC6A16, and PLRG1*, which were shared between the H/FH and FH-CHD groups (Fig. 4). Functional analysis revealed that only 6 of 15 variant genes—*CHUK, EMC10, PLRG1, MAFG, AKAP1*, and TLR5—are potentially related to atherogenesis. The gene description, location, and effect of the variant and remarkable changes in the variant genes are shown in Table 3.

**Table 3:** High-impact variants in the coding regions of seven variant gene intersections between the FH and FH-CHD groups.

Ensembl no. (Transcript ID)	NCBI RefSeq	Symbol	Location (Genomic)	Effects	Alleles (Ref > Alt)	Protein Change	HGVS [19,20] (Genomic) g.∗	HGVS [19,20] (Protein) p.∗	Pathogenicity Scoring (ACMG Criteria) [21]
***FH and FH-CHD: seven genes***									
ENSG00000197063 (ENST00000357736.9)	NM_002359.4	*MAFG*	1365407:17:00	splice acceptor	C > G	–	chr17:g.81923417C > G	p.(?)	· Predicted loss-of-function (splice-site) · VUS
ENSG00000121057 (ENST00000337714.8)	NM_003488.4	*AKAP1*	952021:55:00	splice donor	T > C	–	chr17:g.57120295T > C	p.(?)	· Predicted loss-of-function (splice-site); · VUS
ENSG00000123136 (ENST00000242776.9)	NM_005804.4	*DDX39A*	240184:27:00	stop-gained	G > A	Arg263Ter	chr19:g.14409927G > A	p.(Arg263Ter)	· Predicted loss-of-function (nonsense); · VUS
ENSG00000187554 (ENST00000642603.2)	NM_003268.6	*TLR5*	3718531:58:00	stop-gained	G > A	Arg392Ter	chr1:g.223111858G > A	p.(Arg392Ter)	· Predicted loss-of-function (nonsense); · VUS
ENSG00000058673 (ENST00000367210.3)	NM_001376342.1	*ZC3H11A*	3397457:48:00	frameshift	Ins A	Gln423fs	chr1:g.203847408insA	p.(Gln423fs∗?)	· Predicted loss-of-function (frameshift); · VUS
ENSG00000131778 (ENST00000369258.8)	NM_004284.6	*CHD1L*	1:147264583–147264584	frameshift	Del G	Ala84fs	chr1:g.147264584delG	p.(Ala84fs∗?)	· Predicted loss-of-function (frameshift); · VUS
ENSG00000149311 (ENST00000675843.1)	NM_000051.4	*ATM*	11:108330421–108330424	splice donor	Del GCA	–	chr11:g.108330421_ 108330424delGCA	p.(?)	· Predicted loss-of-function (splice-site); · VUS
***H and FH-CHD: seven genes***									
ENSG00000204178 (ENST00000374343.5)	NM_018202.6	*MACO1*	424149:03:00	frameshift	Ins T	Leu100fs	chr1:g.25448883insT	p.(Leu100fs∗?)	· Predicted loss-of-function (frameshift); · VUS
ENSG00000118900 (ENST00000262376.11)	NM_001079514.3	*UBN1*	81260:26:00	frameshift	Ins A	-/X	chr16:g.4874666insA	p.?	· Predicted loss-of-function (frameshift); · VUS
ENSG00000082068 (ENST00000265107.9)	NM_018034.4	*WDR70*	626757:16:00	frameshift	Ins A	-/X	chr5:g.37605136insA	p.?	· Predicted loss-of-function (frameshift); · VUS
ENSG00000213341 (ENST00000370397.8)	NM_001278.5	*CHUK*	1670086:03:00	frameshift	Ins A	-/X	chr10:g.100204563insA	p.?	· Predicted loss-of-function (frameshift); · VUS
ENSG00000188372 (ENST00000394857.8)	NM_001110354.2	*ZP3*	1274038:06:00	frameshift	Ins G	Val186fs	chr7:g.76441866insG	p.(Val186fs∗?)	· Predicted loss-of-function (frameshift); · VUS
ENSG00000161671 (ENST00000334976.11)	NM_206538.4	*EMC10*	841397:48:00	stop-lost	G > T	∗372Tyr	chr19:g.50482728G > T	p.(Ter372Tyr)	· Predicted altered stop codon (stop-lost); · VUS
ENSG00000063127 (ENST00000335875.9)	NM_014037.3	*SLC6A16*	821787:29:00	stop-lost	T > C	∗34Trp	chr19:g.49306109T > C	p.(Ter34Trp)	· Predicted altered stop codon (stop-lost); · VUS
***H and FH and FH-CHD: one gene***									
ENSG00000171566 (ENST00000499023.7)	NM_002669.4	*PLRG1*	2575656:16:00	frameshift	Ins T	-/X	chr4:g.154539136insT	p.?	· Predicted loss-of-function (frameshift); · VUS

Ins; insert, Del; delete, fs; frameshift, Ter = “termination codon” = stop codon (UAA/UAG/UGA), p.(?) = Uncertain predicted protein effect, p.? = Unknown protein effect, ∗ = stop codon.

Pathogenicity Scoring; Prediction = no segregation, function, population frequency, and several genes are still candidate genes for FH/H/FH-CHD, Variant of Uncertain Significance (VUS) = Insufficient information to conclude whether the variant causes disease [21].

Frameshift variations were found in eight genes—*ZC3H11A*, *CHD1L, MACO1, UBN1, WDR70, CHUK, ZP3,* and *PLRG1*—which disrupted the reading frame, probably resulting in a dysfunctional protein. Stop-gain variations were observed in *DDX39A* at the 6th exon and in *TLR5* at the last intron, which added a premature stop codon, leading to a shortened transcript at amino acid positions 263 and 392, respectively. Stop-lost variations that alter the termination codon and lead to elongated proteins were noted in *EMC10* and *SLC6A16*. *MAFG* harbored a splice-acceptor variant, whereas *AKAP1* and *ATM* harbored splice donor variants.

The descriptions and roles of the six selected variants—*CHUK, EMC10, PLRG1, MAFG, AKAP1,* and *TLR5*—in the context of CVD and other vascular diseases, as reported in previous studies, are described in the Discussion section.

## 4 Discussion

Our recent previous study reported that the expressions of *TRPM2*, *PDLIM5*, *BCL3*, and *GBA* genes in patients with FH can potentially be used as predictive markers of FH-CHD [14]. These genes are involved in chronic inflammation and pathways involved in the CHD pathogenesis. In the present study, using the same volunteers as Prasongsukarn K et al., 2021(14), we attempted to use the alternative advantage of RNA-seq data to identify the potential gene variants in four patient groups and a control group to identify additional probable novel markers. Our findings revealed that the hyperlipidemia, FH, and FH-CHD groups shared several variants among six genes—*MAFG*, *AKAP1*, *TLR5*, *CHUK, EMC10,* and *PLRG1*—which are potentially associated with the progression of CHD. Several previous studies on patients with FH revealed that variants in genes coding for LDL-c receptors and lipid metabolic pathways, such as *APOB*, *LDLR*, *PCSK9*, or *LDLRAP1*, are important risk factors for the diagnosis of FH-CHD [[22], [23], [24], [25]].

However, the gene variants found in our study were not directly associated with major lipid-metabolism pathways. Our findings revealed genes involved in other pathways related to the complex atherogenic process and CHD development, which adds to previous knowledge [4,5,[22], [23], [24], [25]]. We emphasize that these findings are likely to support further in-depth study aimed at clarifying the potential of these variants for use as interdependent predictors of the possibility of FH-CHD progression in hyperlipidemia patients.

RNA-seq analysis has been widely applied to identify and assess the expression of genes in various tissues [6]. Furthermore, this technique also effectively detects transcriptome variants, such as SNPs and short indels, in divergent tissues and species [[8], [9], [10], [11], [12],^26^]. However, in present study, we applied only RNA-seq data to explore the gene variants in the coding regions. We did not screen exon DNA and short neighboring intron sequences as templates to validate the gene variants by Sanger sequencing or gDNA targeted NGS [27]. However, recent advances in NGS have enabled accurate identification of diverse genomic variants, which revolutionizes methods to obtain genomic data [22,24,26,28,29]. Using gene panels and exome and genome sequencing, diverse coding variants can be identified for several disorders [26,30].

In this preliminary study, based on a reference database without any gene variant validation, we attempted to link specific high-impact variants within selected genes—*MAFG, AKAP1, TLR5, CHUK, EMC10*, and *PLRG1* – to their possible roles in atherogenesis and CVD.

*MAFG* encodes for a newly discovered key transcription factor involved in preventing apoptosis of vascular endothelial cells, which is the leading risk factor for atherosclerosis [31]. It also acts as a transcriptional repressor that modulates bile acid and cholesterol metabolism [32]. A previous *in vitro* study on apoptosis of human umbilical vein endothelial cells induced by palmitic acid revealed that *MAFG* and *MAFF* expression could inhibit apoptosis in these cells [31]. The MAFG protein acts synergistically with nuclear factor erythroid 2-like 2 (NFE2L2 or Nrf2) to transcriptionally repress or activate gene functions related to the antioxidant response element (ARE)-dependent pathways in skeletal muscle cells [33]. *MAFF*, *MAFG*, and *NFE2L1* can transcriptionally activate ARE-associated genes cooperatively in arterial endothelial cells, which enhances the antioxidant levels in these endothelial cells, mitigating cell injury and suppressing atherogenesis [31]. Although a splice-acceptor variant was discovered in *MAFG* (Fig. 3, Table 2), no significant changes in the codon or amino acid position were observed. Splice-site variants are alterations of the DNA sequence occurring at the border of an exon and an intron (splice-site), potentially disrupting RNA splicing, resulting in the loss of exons or the inclusion of introns, and altering the protein-coding sequence [29,34].

*AKAP1* encodes a protein belonging to the AKAP family, which binds to the types I and II regulatory subunits of protein kinase A and anchors them to the mitochondrion. Intact mitochondrial homeostasis is pivotal for effective contractile function and metabolism of cardiac muscles. Impaired mitochondrial dynamics contribute to the pathophysiology underlying various CVDs [35]. AKAP1 is potentially related to the cAMP-dependent signal transduction pathway and in regulating RNA to a specific cellular compartment. Therefore, this protein is associated with CVD, mitochondrial function, brain ischemia, hypoxia, EC, HT, lipid metabolism, and myocardial hypertrophy. Deletion of *AKAP1* results in complex II dysfunction. Mitochondrial respiration, or the electron transport chain (ETC), is the main source of superoxide during ischemic and excitotoxic injuries. Therefore, variants in the subunits or assembly factors of the ETC can cause neurodegenerative disorders [36]. In this study, we noted a single splice donor variant in *AKAP1*. Similar to the *MAFG* variant, we found no significant changes in the codon and amino acid positions (Table 2).

*TLR5* encodes a toll-like receptor (TLR) family member, which is crucial for pathogen recognition and activation of innate immunity through the detection of specific pathogen-associated molecular patterns. This gene encodes proteins that recognize bacterial flagellin, the main component of bacterial flagella and a virulence factor. The induction of this receptor triggers the activity of NF-κB, which in turn induces several target genes related to inflammation. The NF-κB family of transcription factors is an important regulator of immune development, immune responses, and inflammation. False regulation of NF-κB is implicated in numerous diseases ranging from inflammatory to immune disorders [37]. Several TLR subfamilies, including TLR2, TLR4, and TLR5, are linked to the initiation and progression of atherosclerotic lesions. In this study, a stop codon variant was noted in *TLR5* that led to translation of a shortened loss-of-function TLR5 protein. This might affect various pathways related to NF-κB activation, affect immune responses to inflammation, and moderate atherogenesis process, as previously reported [37]. In addition, hematopoietic TLR5 deficiency prevents atherosclerotic plaque formation by reducing macrophage accumulation and impairing T-cell responsiveness [38].

*CHUK* is a protein-coding gene that encodes a member of the serine/threonine protein kinase family. The encoded protein is a unit of a cytokine-activated protein complex. In addition, this protein inhibits the fundamental transcription factor NF-κB complex, by phosphorylating sites that induce the inhibitor degradation via the ubiquitination pathway, thereby triggering the transcription factor [provided by RefSeq, Jul 2008]. *CHUK* is one of the detected fatty acid-associated genes, including genes involved in the metabolisms of lipid and/or carbohydrate, or in the arrangement of intramuscular fatty acid accumulation (e.g., *ADIPOQ, CHUK, CYCS, CYP4B1, DLD, ELOVL6, FBP1, G0S2, GCLC, HMGCR, IDH3A, LEP, LGALS12, LPIN1, PLIN1, PNPLA8, PPP1R1B, SDR16C5, SFRP5, SOD3, SNW1,* and *TFRC*) in pigs [39]. Moreover, CHUK plays a pivotal role in hypertension, and hyperlipidemia, which are common risk factors of ischemic stroke (IS). *CHUK* variants rs2230804 and rs3808917 may be associated with elevated blood pressure and lipid levels of IS patients [40].

*EMC10* is a protein-coding gene that encodes 1 of 10 units of the endoplasmic reticulum complex (EMC), which is a highly conserved protein complex associated with the biology of membrane protein (summary by Ref. [41]). EMC10 has several roles, including contributing to membrane insertase activity, positively regulating angiogenesis, promoting endothelial cell proliferation, and facilitating protein insertion into the endoplasmic reticulum (ER) membrane. EMC10 is located in the endoplasmic reticulum membrane and the extracellular region [42]. Furthermore, secreted EMC10 (scEMC10) is upregulated in people with obesity and is correlated with insulin resistance. Overexpressed scEMC10 decreases energy consumption, thus promoting obesity in mice. scEMC10 is a circulating inhibitor of thermogenesis and a potential therapeutic target for obesity and its cardiometabolic complications [43]. EMC10 is produced by monocytes and macrophages derived from bone marrow. It functions as an angiogenic growth factor involved in myocardial infarction repair [44].

*PLRG1* is a protein-coding gene that encodes a core unit of the cell-division cycle 5-like (CDC5L) complex Pleiotropic Regulator 1. The CDC5L complex is part of the spliceosome and is necessary for pre-mRNA splicing. The encoded protein plays a crucial role in alternative splice-site selection. Alternatively, spliced transcript variants encoding multiple isoforms have been observed for this gene (provided by RefSeq, Jan 2011). The *PLRG1* variant reportedly is related to severe congenital human cardiovascular malformations [45]. In a mouse model, *PLRG1* has an essential role as a critical nuclear regulator of p53-dependent cell-cycle progression and apoptosis during both embryonic development and adult tissue homeostasis [46]. Taken together, our findings revealed that the six target gene variants are potentially related to the risk of FH-CHD in patients with hyperlipidemia.

However, this study had some limitations. In this preliminary study, we conducted an exploratory analysis of gene variants with low statistical power (0.53), due to the small genetic sample size and single-center analysis. Further multi-center studies with larger sample sizes and gene variant validation, using either Sanger PCR or gDNA targeted NGS, should be conducted to evaluate the feasibility of key variants for assessing FH-CHD risk.

In addition, the findings of high-impact variations in the six genes and their effects were estimated on the basis of bioinformatic analyses. Further studies involving bioinformatic analyses, variant classification, and functional validation assays are required. This knowledge would help researchers understand the gene function, biological mechanisms of the predicted genes, and variant pathogenicity to identify better predictive, diagnostic, and prognostic methods for premature CHD [34,37].

## 5 Conclusions

Our preliminary study suggested that the variations in the *MAFG, AKAP1, TLR5, CHUK, EMC10, and PLRG1* genes are related to the risk of premature CHD. Further classification and functional validation should be performed to confirm our results and support the use of these gene variants as indicative markers for FH-CHD risk in hyperlipidemia patients.

## CRediT authorship contribution statement

**Wilanee Dechkhajorn:** Data curation, Formal analysis, Investigation, Methodology, Project administration, Validation, Visualization. **Kriengchai Prasongsukarn:** Conceptualization, Data curation, Methodology. **Surachet Benjathummarak:** Formal analysis, Investigation, Methodology, Validation. **Supachai Topanurak:** Supervision, Validation, Visualization. **Yaowapa Maneerat:** Conceptualization, Data curation, Funding acquisition, Methodology, Resources, Supervision, Visualization, Writing – original draft, Writing – review & editing.

## Declaration of competing interest

The authors declare that they have no known competing financial interests or personal relationships that could have appeared to influence the work reported in this paper.

## Data Availability

Data will be made available on request.
