# Supplementary material for: RNA-sequencing based gene variants observed in patients with hyperlipidemia and premature coronary heart disease: A preliminary study
Source: Biochem Biophys Rep. 2026 Jan 24;45:102466. doi: 10.1016/j.bbrep.2026.102466 (PMC12860658; doi:10.1016/j.bbrep.2026.102466)
Supplement: Multimedia component 1 [file mmc1.docx]

**Supplementary Method S1: Command Line Pipeline for RNA-seq Pre-processing and Variant Calling**

This section details the exact command-line workflow used for RNA-seq pre-processing, read-group assignment, variant calling, and variant filtering. All analyses were performed in a Linux environment.

**1. Read Group Assignment and Coordinate Sorting (Picard Tools)**

Read groups were added, and alignment files were sorted by genomic coordinates using Picard Tools (AddOrReplaceReadGroups):

java -jar /usr/local/share/picard-tools/picard.jar AddOrReplaceReadGroups \

I=output.sam \

O=rg_added_sorted.bam \

SO=coordinate \

RGID=ID_NAME \

RGLB=library \

RGPL=illumina \

RGPU=identifier \

RGSM=sample_name

## ****2. RNA-seq Pre-processing for Improved Variant Detection (Opossum)****

Pre-processing of RNA-seq BAM files was performed using **Opossum**, which corrects RNA-seq–specific alignment artifacts and optimizes files for SNP/indel calling (Oikkonen & Lise, 2017):

python Opossum.py \

--BamFile=input.bam \

--OutFile=output.bam

## ****3. Variant Calling (GATK v3 HaplotypeCaller)****

Variants were called using GATK v3 **HaplotypeCaller**, excluding soft-clipped bases and applying a minimum calling confidence threshold of 20:

java -jar /usr/local/gatk3/GenomeAnalysisTK.jar \

-T HaplotypeCaller \

-R /path/to/genome/fasta \

-I recalibrated.bam \

-dontUseSoftClippedBases \

-stand_call_conf 20.0 \

-o Variants_called.vcf

## ****4. Variant Filtering (GATK VariantFiltration)****

Standard hard-filter criteria were applied to remove variants with low quality or strand bias:

java -jar /usr/local/gatk3/GenomeAnalysisTK.jar \

-T VariantFiltration \

-R /path/to/genome/fasta \

-V Variants_called.vcf \

-window 35 \

-cluster 3 \

-filterName Filter -filter "QD < 2.0" \

-filterName Filter -filter "FS > 30.0" \

-o Filtered_variants_called.vcf

## ****Reference****

Oikkonen L, Lise S. Making the most of RNA-seq: Pre-processing sequencing data with Opossum for reliable SNP variant detection. Wellcome Open Res. 2017;2:6.
